# Proteomic Analysis Resolves Distinct Molecular Signatures in Alzheimer’s Disease Brain Across Diverse Racial Groups

**DOI:** 10.1002/alz.092542

**Published:** 2025-01-09

**Authors:** Fatemeh Seifar, Edward J Fox, Anantharaman Shantaraman, Yue Liu, Erica S. Modeste, Duc M Duong, Luming Yin, Marla Gearing, James J. Lah, Joseph S. Reddy, Mariet Allen, Zachary Quicksall, Laura M Heath, Jo Scanlan, Erming Wang, Minghui Wang, Abby Vander Linden, William L. Poehlman, Xianfeng Chen, Saurabh Baheti, Charlotte C.G. Ho, Thuy Nguyen, Geovanna Yepez‐Sisalema, Adriana O Mitchell, Stephanie R Oatman, Xue Wang, Minerva M. Carrasquillo, Alexi Runnels, Thomas G. Beach, Todd Golde, Lisa L. Barnes, Vahram Haroutunian, Bin Zhang, Philip L. De Jager, David A. Bennett, Anna Greenwood, Nilüfer Ertekin‐Taner, Allan I. Levey, Aliza P. Wingo, Thomas S. Wingo, Nicholas T Seyfried, Accelerating Medicine Partnership for Alzheimer’s (AMP‐AD) Diversity Initiative Disease

**Affiliations:** ^1^ Emory University, Atlanta, GA USA; ^2^ Emory University School of Medicine, Atlanta, GA USA; ^3^ Mayo Clinic, Jacksonville, FL USA; ^4^ Sage Bionetworks, Seattle, WA USA; ^5^ Icahn School of Medicine at Mount Sinai, New York, NY USA; ^6^ Mayo Clinic, Phoenix, AZ USA; ^7^ Mayo Clinic, Rochester, MN USA; ^8^ New York Genome Center, New York, NY USA; ^9^ Banner Sun Health Research Institute, Sun City, AZ USA; ^10^ Rush University Medical Center, Chicago, IL USA; ^11^ Columbia University Medical Center, New York, NY USA

## Abstract

**Background:**

Alzheimer's disease (AD) is the most prevalent neurodegenerative disease, yet our comprehension predominantly relies on studies within the non‐Hispanic White (NHW) population. To address this, Accelerating Medicines Partnership in AD (AMP‐AD) aimed to promote inclusivity in multi‐omics AD research, to unravel unique molecular signatures and pathways. The study aimed to provide comprehensive insights into the proteomic landscape of AD across diverse racial groups.

**Method:**

Dorsolateral prefrontal cortex (DLPFC) and superior temporal gyrus (STG) brain tissues were donated from multiple centers (Mayo Clinic, Emory University, Rush University, Mt. Sinai School of Medicine) and were harmonized through neuropathological evaluation, specifically adhering to the Braak staging and CERAD criteria. Among 1105 DLPFC tissue samples (980 unique individuals), 322 were from African American donors, 214 from Latino Americans, 551 from NHW donors, and the rest were from a mixed or unknown racial background. Among 280 STG samples, 86 were African American, 76 Latino American, 116 NHW and the rest were mixed or unknown ethnicity. All tissues were uniformly homogenized and analyzed by tandem mass tag‐based mass spectrometry (TMT‐MS). Following quality control (QC), proteins with more than 50% missing values were removed and iterative principal component analysis was conducted to remove outliers within brain regions. Differential protein expression was conducted within and across races after adjusting for batch using regression‐analysis.

**Result:**

In total, 9,180 proteins from the DLPFC, and 9,734 proteins from STG brain tissues were quantified by TMT‐MS. Further analysis identified approximately 3,000 differentially abundant proteins in AD brains compared to controls within race. The AD‐associated differentially abundant proteins were strongly correlated between DLPFC and STG regions and with CERAD and Braak. Protein levels of MAPT and APP demonstrated equivalent disease‐related elevations in both African American and NHW individuals with AD. Notably, after adjusting for AD, significant differences in protein levels were identified across race.

**Conclusion:**

The proteomics approach employed by AMP‐AD sheds light on the brain proteome in AD across diverse racial groups, emphasizing the importance of inclusive research in establishing robust and generalizable discoveries. The identified proteins here could underscore racial disparities beyond disease association, contributing to a more nuanced understanding of AD pathophysiology.

